# Practitioners’ and researchers’ perspectives on treatment needs and service provision for online child sexual abuse

**DOI:** 10.3389/fpsyg.2025.1602449

**Published:** 2025-09-30

**Authors:** Felipa Schmidt, Ethel Quayle, Sandra Bucci

**Affiliations:** ^1^Division of Psychology and Mental Health, School of Health Sciences, Faculty of Biology, Medical and Health Sciences, Manchester Academic Health Science, University of Manchester, Manchester, United Kingdom; ^2^School of Health in Social Science, University of Edinburgh, Edinburgh, United Kingdom; ^3^Greater Manchester Mental Health NHS Foundation Trust, Manchester United Kingdom

**Keywords:** internet, trauma, online harmful experiences, practitioner, treatment

## Abstract

Online child sexual abuse (OCSA) presents unique and evolving challenges for young people and those supporting them. Although the long-term psychological impacts of OCSA are increasingly recognized, there remains limited guidance for practitioners on how best to assess and respond effectively to its specific features. This study aimed to explore practitioners’ and researchers’ perspectives on current gaps in service provision, assessment practices, and interventions for young people affected by OCSA. An open-ended online questionnaire was emailed to UK-based practitioners (*n* = 10) and researchers with published expertise in OCSA (*n* = 36), of whom seven also had clinical experience (i.e., clinical psychologists, psychiatrists). A total of 46 responses were analyzed using thematic content analysis. Most participants defined OCSA broadly as ‘any sexually abusive act by an adult or peer with a digital or online component’. Both practitioners and researchers emphasized the need for responses that include psychoeducation, trauma-informed care, and safeguarding measures. Participants also highlighted challenges unique to OCSA, such as the permanence of abuse imagery and the perceived agency of victims in online interactions, factors which were seen to require specialized responses. Despite these needs, both groups highlighted gaps in specialist support, practitioner training, and co-ordinated multi-agency responses. The findings highlight the urgent need for further research to develop consensus-based, evidence-informed approaches to OCSA-specific assessment and intervention, ensuring services are equipped to meet the needs of this vulnerable population.

## 1 Introduction

Technology has become a ubiquitous part of children and young people’s lives, with increasingly younger children accessing digital platforms each year. In 2024, 59% of UK children aged 5–7 reported going online to send messages or make voice/video calls (Ofcom, 2024). As children’s everyday experiences are becoming more deeply interwoven with online spaces, so too does their risk of encountering online harms. In the UK, 32% of children aged 8–17 reported seeing something online that they found worrying or distressing (Ofcom, 2024). While not all online experiences result in lasting harm, there is growing concern about the long-term impact of online child sexual abuse (OCSA; Finkelhor et al., 2023; Page et al., 2025). OCSA can be broadly defined as any form of child sexual abuse (CSA) that involves an online element, such as social media or messaging services (ECPAT International, 2016).

In 2021, the UK government published the “Tackling Child Sexual Abuse Strategy”, which emphasized the need for healthcare practitioners to be equipped with the skills to address all forms of CSA, including abuse that occurs online (Home Office, 2021). Since the launch of the strategy, reported cases of OCSA have continued to rise annually (Childlight, 2024; IWF, 2024). The National Center of Missing and Exploited Children (NCMEC) reported a 300% increase of reports of online enticement from 2021 to 2023 (NCMEC, 2025). Equally, recent nationally representative studies both in the US and Australia have highlighted the high prevalence rates of OCSA (Finkelhor et al., 2022; Walsh et al., 2025). In a survey of Australian youth aged 16–24, Walsh et al. (2025) showed that 17.7% had experienced online sexual solicitation before the age 18. The study also revealed that girls were significantly more likely than boys to experience non-consensual image sharing (10.9% vs. 3.8%) and online sexual solicitation (26.3% vs. 7.6%). Qualitative research with young people who have experienced OCSA indicates a range of negative mental health impacts, including anxiety, depression, and suicidal ideation (e.g., Chiu and Quayle, 2022; Joleby et al., 2020). In addition to psychological distress, survivors often report enduring feelings of self-blame and shame, as well as negative consequences for their interpersonal relationships (Schmidt et al., 2023).

Following experiences of OCSA, victims often face significant challenges to accessing appropriate mental health support. These challenges are frequently linked to feelings of shame and self-blame that victims often experience following OCSA, which can inhibit help-seeking behaviors (Joleby et al., 2024). Research has also identified a lack of knowledge and understanding among practitioners regarding the specific nuances of OCSA (Hamilton-Giachritsis et al., 2020; Quayle et al., 2023). Importantly, certain aspects of OCSA differ from offline-only forms of CSA and may require distinct treatment considerations (Knipschild et al., 2025). This gap in understanding is often compounded by the absence of clear guidance for practitioners on how to address young people’s online experiences in clinical settings. In addition, current assessment tools fail to capture the complexities of online behaviors and harms (Dimitropoulos et al., 2022; El-Asam et al., 2021; Schmidt et al., 2024), further limiting practitioners’ ability to identify and respond effectively to the needs of children and young people affected by OCSA.

Given the emerging nature of research in this area, it is important to establish a foundational understanding of how OCSA is currently understood by those working in the field. This includes both practitioners with direct experience supporting young people affected by OCSA, and researchers who have examined its harms and the existing service gaps. An open-ended survey offers a suitable method for generating initial insights into perceived gaps in knowledge and practice. To this end, we sought input from both practitioners and researchers regarding current definitions of OCSA, treatment needs, service provision, and the assessment tools and interventions used. The primary aim of this study was to explore perspectives on treatment needs for young people affected by OCSA, including how these may differ from those associated with offline-only CSA. The secondary aim was to examine practitioners’ views on current service provision, with a focus on assessment processes and the resources required to address identified gaps in care.

## 2 Methods

### 2.1 Participants

We present findings from a study that used a survey with open-ended questions to gather expert insights. Participants were eligible if they met at least one of the following criteria: (a) they were a UK-based practitioner who had worked with at least one individual who had experienced OCSA; and/or (b) they were a UK or non-UK based researcher who had published academic work related to OCSA within the 5 years prior to the study (to ensure that insights were grounded in current evidence and informed by up-to-date research in the field). Given the study required participants with specific expertise in OCSA, purposive sampling was used to ensure that only individuals with relevant professional or academic experience were included. This method was selected to target participants who could provide informed, practice-based or research-informed insights into the current treatment landscape, service gaps, and intervention needs for young people affected by OCSA. Researchers were identified through a review of published academic literature on OCSA, focusing on authors who had contributed to empirical or theoretical work in the field. Practitioners were identified through the professional networks of the research team, including individuals known to have direct clinical experience or involvement in service development related to child sexual abuse or exploitation. This approach allowed us to recruit a sample with the depth of knowledge necessary to address the study’s aims. The study was also promoted via social media, where individuals were invited to contact the research team if they were interested in participating.

### 2.2 Design

The study was approved by the local University Research Ethics Committee (UREC: 022-10369-21699). Participants were asked to respond to questions examining their understanding of how OCSA is defined, the nature of current service provision, and the perceived treatment needs of young people affected by OCSA, particularly in comparison to those affected by offline-only CSA. In addition, participants were asked about the assessment tools and interventions currently used to support this population. A full list of questions can be found in Supplementary Material 1. Both researchers and practitioners received the same survey; however, they were able to skip questions that may not pertain to their expertise.

### 2.3 Procedures

After identifying suitable participants, participants were emailed a link to the survey to complete on the Bristol survey software to share their views on key issues related to current service provisions for OCSA. To mitigate the risk of a low response rate, we contacted a large sample of potential participants (*n* = 314), all of whom met clearly defined eligibility criteria. Previous literature has suggested that clearly defined inclusion criteria can support targeted recruitment and improve the quality of responses (Wu et al., 2022). Thus, a purposive sampling approach was used to ensure that the individuals invited to take part had relevant expertise and experience in OCSA, either as practitioners or researchers. While the final response rate was under 15%, the participants who responded provided rich, in-depth data consistent with qualitative research aims, where depth and relevance of insight are prioritized over statistical representativeness.

### 2.4 Data analysis

Participants were given four weeks to complete the survey. Following this, the responses were downloaded and imported to NVivo for analysis. Content analysis was used to analyze the survey responses, following the approach outlined by Vaismoradi and Snelgrove (2019). This method was selected as it is well suited to systematically examining qualitative data with the aim of identifying patterns, meanings, and categories within open-text responses. The analysis process involved several stages. First, responses were read and re-read to ensure familiarization with the data. Words, phrases, and paragraphs conveying similar meanings were then grouped into thematic categories for each survey question. These categories were developed inductively, meaning that themes were derived directly from the content of participants’ responses rather than being imposed *a priori*. Once thematic categories were developed, they were quantified by frequency of occurrence to identify the most expressed views, while still preserving attention to less frequent but conceptually important responses. This process informed the development of summary statements that captured key themes across responses. Data were managed and analyzed using Microsoft Excel, which was used to organize responses by question, code excerpts, and track the frequency of emerging categories. This tool provided an accessible and flexible means of conducting and auditing the analysis.

To enhance the trustworthiness of the study, we took several steps. First, we provided transparency around sampling, recruitment, and analytical procedures. Second, themes were generated inductively through a structured content analysis process, and the frequency with which certain responses occurred was reported descriptively to indicate patterns within the sample, not to imply generalizability to a broader population. In line with qualitative research conventions, we interpret our findings through the lens of transferability rather than generalization, recognizing that our results may offer useful insights for similar professional and service contexts.

## 3 Results

A total of 314 practitioners and researchers were invited to participate in the study, of whom 46 completed the open-ended survey. The sample included 10 practitioners, comprising clinical psychologists (*n* = 3), a forensic psychologist (*n* = 1), social workers (*n* = 4), and mental health practitioners (*n* = 2). The remaining 36 participants were researchers with published expertise in OCSA, drawing from fields such as psychology, criminology, and cyber security. Of these, seven also held clinical qualifications or roles, including registered social workers, psychiatrists, or clinical psychologists. Participants reported a wide range of experience working in the field of OCSA, from one year to over 20 years, with half having >10 years of relevant experience. The researcher group was internationally diverse, including 22 participants from the United Kingdom, nine from European countries, eight from North America, and seven from additional countries (Australia, China, Thailand and Chile each).

Table 1 presents a list of statements derived from participants’ responses to the key questions explored in this study: (i) definitions and conceptualizations of OCSA; (ii) perceived treatment needs of young people who have experienced OCSA; (iii) distinctions between treatment needs for OCSA and offline-only CSA; (iv) the extent to which current service provisions meets young people’s needs; (v) resources available to practitioner’s within services; (vi) current assessment frameworks used in practice; and (vii) existing interventions for young people affected by OCSA. Participants were also invited to share their perspectives on designing an ideal service model and to describe how they would support young people who have experienced OCSA. The themes generated from the content analysis of this question are presented in Table 2, ranked from most to least frequent. The table also includes a brief description of each theme.

**TABLE 1:** List of thematic statements.

Statement	No. participants
**Definition of OCSA**	
Any sexually abusive act by an adult or peer with a digital or online component	37
Image-related abuse	31
Exchange of self-produced sexual content (e.g., images, videos, livestreams)	19
Any non-consensual act, with or without the use of force	18
Sexual conversations conducted online	16
Offenses involving minors (individuals under 18 years old)	16
Online abuse that may or may not lead to in-person abuse	11
Sexual coercion, deception or manipulation by an adult or peer through online means	10
Online abuse involving compensation for the young person or perpetrator	6
**Perceived treatment needs**	
Empathy and emotional support (e.g., individual/group therapy, counseling, personal support system) to address feelings of shame, guilt, self-blame, aloneness, anxiety, depression	25
Trauma-informed interventions (e.g., post-traumatic therapy, trauma-focused cognitive behavioral therapy)	19
Psychoeducation or training to help young people stay safe online in the future	13
Individualized assessment and person-centered intervention considering broader life context (current and wider circumstances)	13
Support in coping with the permanence and resurfacing of abuse materials	10
Safeguarding practices, including working with relevant authorities to protect the victim and removal of online content	9
Access to appropriate therapeutic interventions (e.g., psychotherapy, family therapy, cognitive behavioral therapy)	8
Recognition and treatment of potential physical impacts related to OCSA	2
**Differences in treatment needs compared to offline CSA**	
Revictimisation and lack of closure due to the permanence and visibility of abuse materials online	23
Greater need for psychoeducation for both young people and parents on online safety and safe online relationships	15
Complexity arising from integration online and offline worlds in young people’s lives	15
There is little or no difference between OCSA and offline CSA (therapeutic response should be person-centered, psychosocial focus to address current and ongoing needs)	15
Increased perception of the young person’s agency in the abuse, leading to heightened guilt, self-blame, shame and negative feelings about self	13
**Current service provisions**	
General lack of knowledge and understanding about OCSA among service providers	23
Inconsistent, limited, or under-resourced specialist services, or inadequate service/specialist provision available, often described as a ‘postcode lottery’	9
OCSA is often treated the same as offline abuse or minimized as a general ‘online problem’ or law enforcement issue (i.e., a police matter)	8
Inadequate assessment practices, including failure to ask directly about online abuse and OCSA	5
Limited psychoeducation is provided on online safety and recognizing unsafe relationships online	5
Services are considered adequate	4
**Resources needed within service provisions**	
Greater access to training and current research on OCSA for practitioners	10
Availability of appropriate, tailored interventions (e.g., psychoeducation, bespoke individually tailored intervention, 1:1 or group therapeutic work and support)	9
Increased peer support and collaboration with specialist charities or partner organizations	8
Use of existing safeguarding plans	2
Integration of routine assessment tools in service delivery	1
**Current assessment tools**	
Assessment mirrors usual general assessments, which might include a disclosure of abuse emerging during assessment of another presentation	8
Use of standardized frameworks and assessments specifically for OCSA (e.g., NSPCC, AIM)	5
Individualized assessments tailored to each young person’s needs	4
Formulation-based approaches informed by information gathering sessions with young people and their families	2
Assessment is tailored to OCSA without the use of standardized tools or frameworks but with consideration of technology and its role in the abuse	1
**Current interventions**	
Person-centered individual or group therapeutic work addressing emotional responses like shame and embarrassment	10
Trauma-focused intervention work	9
Research and education including development of evidence-based practice guidance	4
Crisis intervention and safeguarding responses	4
Joint work with relevant other authorities and agencies	4
Medical interventions, including health checks	1

OCSA, online child sexual abuse; NSPCC, national society for prevention of cruelty to children; AIM, Assessment, Intervention, Movement.

**TABLE 2:** Summary of the ideal service design recommendations.

Theme	Description
Addressing shame, self-blame, and guilt	Focus on reducing shame, self-blame, and guilt through self-care, psychoeducation, and support for parents/guardians (*n* = 11).
Practitioner training on OCSA	Need for specialized training on the impacts of OCSA and how to work effectively with survivors (*n* = 9).
Multidisciplinary integration and collaboration	Importance of liaising with specialist services and promoting cross-agency knowledge sharing (*n* = 8).
Research and evaluation of services	Call for more research into young people’s service experiences and regular evaluation of current services (*n* = 3).
Service communication and accessibility	Emphasize clear communication that services support online abuse-related issues. (*n* = 2)
Peer support and tailored care	Encourage peer support (*n* = 2) and provide individually tailored care to meet each young person’s needs (*n* = 3).

Overall, eleven participants highlighted the importance of addressing common emotional responses, including shame, self-blame, and guilt. Suggested strategies included integrating components focused on self-care, providing psychoeducation, and offering support for parents/guardians. Nine participants highlighted the need for specialized training for practitioners, specifically covering the unique impacts of OCSA and how to best engage with and support survivors. Eight participants discussed the importance of multi-disciplinary integration and collaboration. This included working closely with existing specialist services and promoting knowledge-sharing across agencies to ensure comprehensive, co-ordinated care. In relation to service development, and in creating the “ideal service”, three participants stressed the need for ongoing research into young people’s experiences with services, as well as routine evaluation of existing provisions to understand the need and ensure they are meeting service users’ needs. Additional suggestions included clearly communicating to young people that support and treatment for online experiences are available, providing opportunities for peer support, and ensuring that services are flexible, individualized, and responsive to each young person’s specific needs (Table 2).

Table 1 presents the thematic categories generated through content analysis of participants’ responses to the open-ended questionnaire. The themes reflect patterns of meaning identified across the data set and are organized by frequency, indicating how commonly each theme was mentioned. The most endorsed definition of OCSA was “any sexually abusive act by an adult or peer with a digital or online component.” While there was broad agreement on this general framing, the variety of responses reflects considerable variation in how participants conceptualized OCSA, reflecting its multifaceted nature. Participants identified a range of therapeutic and support needs, with the most frequently cited being “empathy and emotional support to address feelings of shame, guilt, self-blame, aloneness, anxiety, depression”. A consistent pattern emerged highlighting the importance of holistic, trauma-informed, and tailored care, with a focus on both emotional recovery and practical safety. Key differences between OCSA and offline CSA were noted, with the most frequently mentioned being the experience of revictimisation and lack of closure due to the permanence and visibility of abuse materials online. While some participants (*n* = 15) believed that the therapeutic response should not differ between OCSA and offline CSA, reinforcing the value of person-centered care, others highlighted the unique challenges posed by OCSA. In particular, the persistence and visibility of online abuse materials were seen as distinctive features that can lead to revictimisation and ongoing distress, thereby warranting adapted and specialized therapeutic strategies. Significant concerns were raised about the adequacy of current services. Participants reported that many existing service structures are not well equipped to recognize or respond effectively to OCSA, highlighting a systemic gap in care. The findings highlight a pressing need for workforce development, structured tools, and greater collaboration with specialist services to strengthen responses to OCSA.

## 4 Discussion

The study presents important insights into the perceived treatment needs of young people affected by OCSA, which supports existing concerns within the literature regarding gaps in current service provision. Participants responses echoed previous findings by highlighting the lack of tailored interventions and specialized service provision (Quayle et al., 2023), as well as the limited availability of appropriate assessment tools to address the unique challenges associated with OCSA (Schmidt et al., 2023). These challenges included the permanence of abuse images, the complex interplay between young people’s online and offline lives, and feelings of self-blame arising from perceived responsibility for the abuse.

Research has shown the importance of fostering an open and flexible environment when engaging young people in conversations about their online experiences (Biddle et al., 2022). Consistent with this, the current study highlighted the importance of empathy and supportive engagement in addressing the treatment needs of young people presenting to clinical services following experiences of OCSA. While several identified needs, such as empathy, emotional support, psychoeducation, and trauma-informed interventions, align with treatment approaches for offline CSA and broader trauma therapy (e.g., Cummings et al., 2012; Sweeney et al., 2018), this study also highlighted needs that may require more specialized responses in the context of OCSA. This includes recognizing that OCSA may involve physical elements of harm, which may differ in nature or context from those typically observed in offline-only CSA. Additionally, it involves addressing the anxiety and fear associated with the permanence and potential resurfacing of abusive images online. This latter feature is a unique and often distressing aspect of OCSA, contributing to re-traumatization and complicating recovery, as shown in prior research (e.g., Finkelhor et al., 2023). In addition, this study reinforces existing concerns about the limited availability and capacity of specialist services equipped to support young people affected by OCSA. Participants noted a general lack of professional knowledge and understanding of OCSA, which is consistent with findings from research (Quayle et al., 2023). Together, these findings highlight the need for targeted training to increase practitioner’s confidence, competence, preparedness and knowledge in addressing the specific challenges associated with OCSA (El-Asam et al., 2021). Recent initiatives have sought to develop frameworks to support practitioners in recognizing and responding to online harms, including OCSA, during clinical assessment (Biddle et al., 2022; Tully et al., 2025). For example, a recent Australian study provided practical guidance on how practitioners can identify and address signs of OCSA in their work (Tully et al., 2025). These efforts reflect a growing international call for clearer guidance and structured assessment approaches in this area. However, further empirical research is needed to evaluate and validate such tools, particularly within the UK context.

The current study also highlighted the challenges in establishing a clear and consistent definition of OCSA. While most participants (80%) defined OSCA as “any sexually abusive act by an adult or peer with a digital or online component”, others offered narrower definitions focused on specific behaviors such as “image-related abuses”, or exchange of “self-produced sexual content”. Some participants also emphasized the importance of including sexual coercion or elements of blackmail as defining features. Although there was general agreement on certain aspects, the findings highlight the lack of a single, universally accepted definition of OCSA. This definitional ambiguity is echoed in the wider literature, where inconsistencies across studies complicate efforts to accurately measure prevalence and monitor trends over time (Page et al., 2025). For practitioners, uncertainty around what constitutes OCSA can make it difficult to identify, assess, and respond appropriately to affected young people (Quayle et al., 2023). These challenges are compounded by the lack of validated tools for routinely assessing OCSA in clinical or safeguarding contexts (Quayle et al., 2023; Zeyzus Johns et al., 2024). Without clear definitions and assessment frameworks, practitioners may feel less confident in asking about OCSA or knowing how to respond to disclosures.

The current study revealed some divergence in participants’ views regarding whether the treatment needs of young people affected by OCSA differ from those affected by offline-only CSA. While several participants reported that the treatment needs are broadly similar across both contexts, others highlighted the need for OCSA-specific considerations. These included addressing the psychological impact of the continued circulation of abusive images online (i.e., the permanence of abusive images) and the potentially increased perception of agency in the abuse, which may amplify feelings of shame and self-blame. Such findings are evidenced in previous qualitative research, which has shown self-blame and shame as common emotional responses among survivors of both OCSA and offline CSA (e.g., Joleby et al., 2020; Kennedy and Prock, 2016). However, unique to OCSA is the victim’s fear and worry related to the enduring presence and potential redistribution of abusive images, which can contribute to ongoing distress and re-traumatization (Joleby et al., 2020). These findings highlight the need for nuanced, trauma-informed approaches that recognize both shared and distinct psychological impacts associated with different forms of CSA.

The findings of the study should be considered in light of some limitations. First, while the response rate to the survey was relatively low (14.6%), this is not unusual for online surveys targeting specialized professional groups. Although the study is not statistically generalizable, its purposive sampling approach ensured that participants had relevant expertise, and the data generated were rich and informative. Notably, several of the themes identified in this study are consistent with findings reported in other research on service provision and professional responses to CSA. This alignment suggests that the findings may be transferable to similar clinical and research contexts and may contribute meaningfully to the development of future training, service models, and research in OCSA. Second, the sample included more researchers than practitioners and the analysis did not differentiate between the individual group’s responses. Therefore, views presented here are not fully representative of all practitioners working with young people affected by OCSA. Third, while this study focuses on practitioners’ and researchers’ views, incorporating the voices of young people with lived experience of OCSA would offer a more comprehensive understanding of treatment needs. Finally, the reliance on self-reported data collected through an open-ended survey limited the depth of responses when compared to interviews or focus groups.

The findings of this study have several important implications for research, practice, and policy. First, they highlight the need for a comprehensive empirical program to further investigate the treatment needs of young people affected by OCSA and how it differs from those affected by offline-only CSA. Such research would provide the foundation for developing evidence-based best practice guidelines, targeted training materials, and specialist clinical frameworks that address the specific and evolving treatment needs of OCSA. The findings also point to gaps in current service provision and practitioner confidence, underscoring the importance of equipping frontline staff with the tools and knowledge required to respond effectively to OCSA in clinical and safeguarding contexts. In addition, the study has implications for service commissioning and design. The perspectives gathered suggest a need for dedicated care pathways and better integration of OCSA-specific considerations within existing child and adolescent mental health services. Multi-agency collaboration is also essential, particularly in aligning mental health, wider digital harms, child protection, and education sectors to ensure a coherent response. Furthermore, the study reinforces the importance of developing early intervention and prevention strategies, including incorporating OCSA-related content into youth services and digital safety education. At a policy level, these findings contribute to the growing recognition of OCSA as a distinct form of harm requiring tailored and well-coordinated responses. Overall, this study provides a foundation for advancing clinical, service, and policy responses to OCSA and highlights the urgency of building specialist capacity across systems and services that support vulnerable young people.

Future research should prioritize the co-development of practice-informed guidelines, including input from young people with lived experience, to inform treatment pathways and improve the quality, relevance, and consistency of support available to this vulnerable population. In addition, future work should focus on the development and evaluation of tailored psychological interventions specific to OCSA, as well as comparative studies examining how the treatment needs of young people affected by OCSA differ from those affected by offline-only CSA. Future research on practice-informed guidelines needs to be cognizant of the evolving digital advancements (e.g., generative AI, virtual reality) and how they might impact the treatment needs of OCSA. Research should also explore the training needs of practitioners across mental health, education, and safeguarding sectors to ensure that the workforce is adequately prepared. Further investigation is needed into how young people currently access services, including potential barriers in referral pathways, and the feasibility of digital or hybrid models of care. Finally, longitudinal studies are essential to understand the evolving impact of OCSA over time and to inform developmentally appropriate, sustained support.

## 5 Conclusion

The findings of this report present key insights from an open-ended questionnaire exploring how services currently address OCSA. The study highlights notable gaps in service provision, including the limited availability of tailored assessment tools, minimal use of OCSA-specific interventions, and a lack of practitioner training in this area. Importantly, the responses highlight the need for clinical approaches that consider the unique aspects of OCSA, such as the enduring nature of abusive images and the integration of online and offline experiences in young people’s lives. The findings also highlight the importance of equipping practitioners with training that explicitly addresses these nuances, as well as ensuring safeguarding procedures and referral pathways are adapted to recognize and respond to OCSA. As an initial next step to inform practice-informed guidelines, a Delphi study is suggested to reach expert consensus on best practices in both treatment provision and assessment for young people affected by OCSA. This would be a critical step toward developing coherent, evidence-based frameworks and ensuring practitioners are adequately equipped to respond to this evolving area of need. By building on these initial findings, a Delphi study would provide valuable insights to inform the development of more responsive and effective service models.

## Data Availability

The datasets are not readily available because they contain information that could compromise research participant privacy. Requests to access the datasets should be directed to the corresponding author of this manuscript.
