# Supplementary material for: Practitioners’ and researchers’ perspectives on treatment needs and service provision for online child sexual abuse
Source: Front Psychol. 2025 Sep 30;16:1602449. doi: 10.3389/fpsyg.2025.1602449 (PMC12518301; doi:10.3389/fpsyg.2025.1602449)
Supplement: Supplementary file 1 [file Table_1.docx]

**Supplementary Material 1**: Full list of questions asked to participants

|  | Question |
| --- | --- |
| 1 | How would you define online childhood sexual abuse? |
| 2 | Based on your knowledge and experience, what do you perceive to be the treatment needs of young people who have been sexually abused online? |
| 3 | Based on your knowledge and experience, how might the treatment needs of young people who have been sexually abused online, be different to those of young people who have experienced offline sexual abuse? |
| 4 | Based on your knowledge and experience, how well does current service provision meet the needs of young people exposed to sexual abuse online? |
| 5 | What resources within your service can you draw upon to help meet the needs of these young people? |
| 6 | A young person presents to your service and identifies they have met someone online. What would your initial response be? Thinking about your current service provision, what do you have to offer this person? |
| 7 | A young person presents to your service and identifies they have been sexually abused online. What would your initial response be? Thinking about your current service provision, what do you have to offer this person? |
| 8 | How are young people who have been exposed to sexual abuse online assessed in your service? |
| 9 | Could you give any specific examples of interventions for young people who have been sexually abused online that you, your organisation or research group have been involved in? |
| 10 | Based on your knowledge and experience, if you were to design a new service, what would you do differently when working with young people that have been exposed to online sexual abuse? |
